# The *Pseudomonas syringae* Type III Effector HopF2 Suppresses Arabidopsis Stomatal Immunity

**DOI:** 10.1371/journal.pone.0114921

**Published:** 2014-12-11

**Authors:** Brenden Hurley, Donghyuk Lee, Adam Mott, Michael Wilton, Jun Liu, Yulu C. Liu, Stephane Angers, Gitta Coaker, David S. Guttman, Darrell Desveaux

**Affiliations:** 1 Department of Cell & Systems Biology, University of Toronto, 25 Willcocks St., Toronto, Ontario, Canada; 2 Department of Plant Pathology, University of California Davis, Davis, CA, United States of America; 3 Leslie Dan Faculty of Pharmacy, Department of Biochemistry, University of Toronto, Toronto, Ontario, Canada; 4 Centre for the Analysis of Genome Evolution & Function, University of Toronto, Toronto, Ontario, Canada; Virginia Tech, United States of America

## Abstract

*Pseudomonas syringae* subverts plant immune signalling through injection of type III secreted effectors (T3SE) into host cells. The T3SE HopF2 can disable Arabidopsis immunity through Its ADP-ribosyltransferase activity. Proteomic analysis of HopF2 interacting proteins identified a protein complex containing ATPases required for regulating stomatal aperture, suggesting HopF2 may manipulate stomatal immunity. Here we report HopF2 can inhibit stomatal immunity independent of its ADP-ribosyltransferase activity. Transgenic expression of HopF2 in Arabidopsis inhibits stomatal closing in response to *P. syringae* and increases the virulence of surface inoculated *P. syringae.* Further, transgenic expression of HopF2 inhibits flg22 induced reactive oxygen species production. Intriguingly, ADP-ribosyltransferase activity is dispensable for inhibiting stomatal immunity and flg22 induced reactive oxygen species. Together, this implies HopF2 may be a bifunctional T3SE with ADP-ribosyltransferase activity required for inhibiting apoplastic immunity and an independent function required to inhibit stomatal immunity.

## Introduction

*Pseudomonas syringae* is a prominent microbial inhabitant of freshwater ecosystems that has evolved to proliferate and causes disease in plants [1]. In association with plants *P. syringae* survives as an epiphyte on the leaf surface and subsequently enters plant tissues via wounds or natural openings such as stomata. Inside plant tissues *P. syringae* proliferates extracellularly in the apoplastic spaces of leaf or vascular tissues and severely compromises plant fitness. The plant innate immune system limits infection by preventing the invasion of the apoplastic space by epiphytes as well as by compromising the proliferation of the pathogen post-invasively. Both levels of plant immunity are activated by the recognition of microbe-associated molecules termed microbial- or pathogen-associated molecular patterns (MAMPs or PAMPs) by membrane-associated pattern recognition receptors (PRRs); a process termed PAMP-triggered immunity (PTI). Pre-invasively, PTI activates a stomatal immune response resulting in stomatal closure which limits microbial ingress into the apoplastic space [2]. Post-invasively, PTI results in transcriptional reprogramming and the secretion of antimicrobials and cell wall associated compounds such as callose into the apoplastic space [3]. Both levels of PTI involve the production of reactive oxygen species (ROS) and the activation of MAP kinase signalling cascades [3], [4]. Although these two levels of PTI are effective at preventing the invasion and proliferation of most microbes, pathogens such as *P. syringae* have evolved strategies to overcome both pre- and post-invasive PTI.

Many pathovars of *P. syringae* produce the phytotoxin coronatine which enables them to reopen stomata closed by PTI, thereby allowing the pathogen to invade the plant tissue [2]. As a result, *P. syringae* strains that have been deprived of coronatine production do not efficiently invade and proliferate in the apoplast when surface inoculated [2]. In addition to disabling stomatal immunity, coronatine also inhibits apoplastic immunity through suppression of salicylic acid mediated defenses [5], [6]. Apoplastic virulence of *P. syringae* also heavily relies on the type III secretion system (T3SS) to inject effector proteins into host cells. A major function of type III secreted effectors (T3SEs) is the suppression of PTI by targeting PRR complexes and/or downstream signalling components [7], [8]. Although the role of T3Es in apoplastic PTI suppression is well established, evidence of their ability to influence stomatal immunity is limited. Recognition of the T3SE AvrRpt2 by the *Arabidopsis thaliana* (hereafter Arabidopsis) RPS2 NB-LRR (NLR) protein activates effector-triggered immunity (ETI) that is accompanied by stomatal closure [2], [9]. More recently, the *P. syringae* T3SE HopM1 has been demonstrated to suppress PAMP-induced stomatal closure when expressed transgenically in Arabidopsis demonstrating that T3SEs can influence stomatal immunity [10]. In addition, the T3SE HopX1 can promote the growth of COR-deficient *P. syringae* pv. tomato DC3118 (*Pto* DC3118) and also promote stomatal opening [11].

The HopF T3SE family is broadly distributed among pathovars of *P. syringae*. HopF1 from the bean pathogen *P. syringae* pv. phaseolicola 1449B is recognized in bean cultivars expressing the R1 resistance (NLR) protein [12], [13]. The crystal structure of HopF1 revealed that it adopts a mushroom-like shape with “head” and “stalk” subdomains [14]. The head subdomain possesses limited structural similarity to ADP-ribosyltranferases such as Diphtheria toxin. This similarity was used to predict functional residues that were demonstrated to be required for virulence and avirulence functions of HopF1 on susceptible and resistant bean plants, respectively [14]. Equivalent residues have also been demonstrated to be required for the virulence functions of HopF2 from *Pto* DC3000 (hereafter HopF2) in tomato and Arabidopsis [15], [16]. These virulence functions likely involve interaction and modification of the PRR-associated receptor-like kinase BAK1, MAP kinase kinases and the PTI/ETI regulator RIN4 [15]–[17]. HopF2 has been demonstrated to ribosylate MKK5 and RIN4 *in vitro* and requires RIN4 for apoplastic growth promotion of *P. syringae* in Arabidopsis [15], [16]. HopF2 has also been demonstrated to suppress the ETI-response induced by the T3SE AvrRpt2 [16]. Importantly, all of the HopF2 activities described above require intact catalytic residues of the head-domain.

Here we demonstrate that HopF2 can suppress stomatal immunity in Arabidopsis. Unlike the functions ascribed to HopF2 thus far, suppression of stomatal immunity does not require catalytic residues of the head-domain. These results indicate that HopF2 has distinct molecular functions for the manipulation of apoplastic and stomatal immunity.

## Materials and Methods

### Bacterial strains, Plasmids and Plant Materials and Methods

*Arabidopsis thaliana* ecotype Col-0 were routinely grown with 9 h of light (130 microeinsteins m^−2 ^s^−1^) and 15 h of darkness at 22°C in Sunshine Professional Growing Mix LC1 (SunGro, Canada) soil supplemented with 20∶20∶20 fertilizer.

*Pseudomonas syringae* strains carrying HopF2*_Pto_*_ DC3000_ with an ATG start codon (hereafter HopF2) on the multicopy plasmid pBBR1 MCS-2 were constructed as previously described [16]. Transgenic Arabidopsis expressing HopF2 from the dexamethasone inducible pDB vector were constructed as previously described [16], [18]. In the text, Arabidopsis mutant *rps2* refers to *rps2-101c; rin4* to *rin4* T-DNA insertion line; *mpk3* (SALK_151594) and *mpk6* (SALK_073907) to T-DNA insertion lines [19]–[23]. Transgenic Arabidopsis expressing dexamethasone inducible HopF2 in a *rps2/rin4* mutant background were constructed by crossing T3 transgenic Arabidopsis expressing HopF2 from the dexamethasone inducible pDB vector into the *rps2/rin4* background [24]. Transgene expression was confirmed by immunoblot, and *rps2/rin4* loci confirmed by sequencing. Dexamethasone treatment was conducted as previously described [25].

### *Pseudomonas syringae in planta* Growth Assay and Measurements of Stomatal Aperture

Growth assay with *Pto* DC3000 or *Pto* DC3118 were performed on 4 week old Arabidopsis [26], [27]. Surface inoculation was performed by dipping Arabidopsis grown in mesh enclosed pots into *P. syringae* suspensions according to Melotto *et al*. [2]. Bacterial *in planta* growth assay by syringe inoculation was performed according to Wilton *et al.* [16], [28]. Stomatal apertures were measured according to Liu *et al.* for *P. syringae* induced closure and re-opening [29]. ABA-induced stomatal closure was analyzed on true leaves of two week old Arabidopsis leaves. Leaves were floated on stomata opening buffer (5 mM KCl, 50 uM CaCl2, 10 mM MES-KOH, pH 6.15) overnight and incubated in light (100 microeinsteins m^−2 ^s^−1^) for four hours to induce uniform stomatal opening. A final concentration of 10 µM ABA was added to each well and incubated for 2 hours. Stomatal apertures were then measured according to Liu *et al.* [29].

### Isolation of High Molecular Weight HopF2 Complexes

HopF2 complexes were isolated from 4 week old *Arabidopsis.* HopF2-HA transgenic Arabidopsis rosettes were snap frozen in liquid nitrogen then homogenized in of ice-cold extraction buffer (1 g FW: 2 ml EB) composed of 50 mM HEPES-KOH (pH 7.5), 50 mM NaCl, 2 mM EDTA, 1 mM DTT, 0.2% Triton-X 100, 0.1 mg/ml dextran and 1∶100 (v/v) plant protease inhibitor (Sigma, P9599). Homogenates were clarified by centrifugation at 4°C and 10,000×g for 10 min to remove cell debris and insoluble particulates, and supernatants reserved as clarified extract.

Clarified extracts (4 ml) were injected onto a pre-packed 16/60 Sephacryl S300 HR column (GE, 17-1167-01) and developed at 1.0 ml/min in of 50 mM HEPES-KOH (pH 7.5), 150 mM NaCl, 1 mM DTT, 0.01% Triton-X 100, 0.1 mg/ml dextran using a FPLC (GE). Fractions were collected from the void volume and every third fraction analyzed by immunoblot for HopF2-HA. In subsequent runs, high molecular weight fractions containing HopF2-HA were pooled, concentrated with an Amicon Ultra-15 (10 k NMWL), and subjected to immunoprecipitation with magnetic anti-HA resin (Miltenyi, µMACS anti-HA, 130-091-122).

Pooled concentrated fractions (∼10 ml) were incubated with 150 ul µMACS anti-HA resin for 3 hours, then immobilized on a paramagnetic column (Miltenyi, M column, 130-042-801) For immunoblot analysis of purifications, immobilized resin was washed with 10 bed volumes low salt wash (LSW, 50 mM HEPES-KOH (pH 7.5), 50 mM NaCl, 1 mM DTT) and 10 bed volumes no salt wash (NSW, 50 mM HEPES-KOH (pH 7.5), 1 mM DTT), then eluted over three bed volumes with 0.1 M NH_4_OH. Samples for proteomic analyses were washed three times with 10 bed volumes PBS to remove detergent and eluted over three bed volumes with 250 µg/ml HA peptide in PBS.

### Proteomic Methods

Proteins in HA peptide eluates were precipitated by addition of trichloroacetic acid to a final concentration of 20% (w/v), incubated overnight at 4°C and pelleted by centrifugation (30 min, 20,000×g). Pellets were washed once with 10% TCA, three times with ice cold acetone then solubilized in 50 mM ammonium bicarbonate and digested with trypsin. Spectra were developed using a Thermo Scientific LTQ-XL MS/MS. MS/MS spectra were analyzed and protein identities assigned using SeQuest against the Arabidopsis UniProt FASTA database [30]. Protein identities were verified by Petide/Protein Prophet using a probability score of 0.95 [31].

### MAPK Phosphorylation Status

Arabidopsis seedlings were grown for 11 days on 0.5x MS salts with Gamborg’s vitamins (M0404; Sigma) media solidified with 0.8% agar then transferred to six well plates (six seedlings per well) containing 3 ml of liquid medium containing 0.5x MS salts with 30 µM DEX (for +DEX treatment) or 0.5xMS salt (–DEX). Seedlings were gently shaken in 12 h light, 12 h dark cycle at day/night temperature regime of 22°C/18° for. After 24 h, flg22 peptide was added to a final concentration of 1 µM. Shaking was maintained for 20 minutes then seedlings were harvested and snap frozen in liquid nitrogen. Frozen tissue was homogenized in 100 µl of extraction buffer (100 mM HEPES, pH 7.5, 5 mM EDTA, 5 mM EGTA, 2 mM dithiothreitol, 10 mM Na_3_VO_4_, 10 mM NaF, 50 mM ß-glycerolphosphate, 1 mM phenylmethylsulfonyl fluoride, and 10% glycerol, 1% (w/v) polyvinylpolypyrrolidone). After centrifugation at 13,000 rpm for 30 min at 4°C, supernatants were reserved as clarified extract. Protein concentrations of clarified extracts were determined using a Bradford assay (BIO-RAD, Hercules, CA, USA) and twenty µg of total protein was separated by SDS-PAGE and immunoblotted.

### SDS-PAGE and Immunoblots

Proteins samples were heated in Laemmli sample buffer for 5 min at 95°C and resolved by 12% SDS-PAGE and transferred to nitrocellulose membranes. Blots were probed with either 1∶20,000 α-HA antibodies (Roche), 1∶10,000 α-RIN4 antibodies (Courtesy of G. Coaker), or anti-phospho-p44/42 MAPK (1∶2000, Cell Signaling Technology, Danvers, MA, USA). Immunoreactive bands were visualized using horse radish peroxidase-conjugated secondary antibodies and detected via chemiluminescence (Amersham ECL, GE, USA).

### Quantification of Callose Deposition

Callose deposition was induced by infiltration with *Pto* DC3000Δ*hrcC* at an OD_600_ = 0.2 (1×10^8^ cfu/ml) [28], [32], [33]. Leaf samples were cleared 24 hours post inoculation with an alcoholic lactophenol solution, rinsed in 50% ethanol, and rinsed in water as described [32]. Cleared leaves were stained with 0.01% (wt/vol) aniline blue in a solution of 150 mM K_2_HPO_4_, pH 9.5, for 30 min. Callose deposits were visualized by fluorescence microscopy (UV filter; Leica, MZ16F), and captured using OpenLab software (PerkinElmer). Callose deposits was quantified using Image J software [34].

### ROS Generation Assay

Measurement of ROS production was performed using a modified version of the luminol-based assay as described [35] A series of 20 leaf discs (diameter 4 mm) were taken from the leaves of 4 week-old Arabidopsis. Leaf disks were divided with 10 disks placed in each of two wells of a 96-well plate and washed with 200 µl of sterile water for 20 hours. The water was removed and replaced with 100 mM Tris-HCl (pH 8.0) containing 20 µg/ml horseradish peroxidase and 34 µg/ml luminol. Each matched pair of wells from a single plant was treated with either water or 2 µM flg22 peptide. The luminescence was measured for a 2 second interval every 2 minutes for a total of 60 minutes on an Infinite M1000Pro microplate reader (TECAN Group Ltd.). Each treatment was performed on three individual plants.

## Results

### Analysis of HopF2 protein complexes

**Figure 1:**
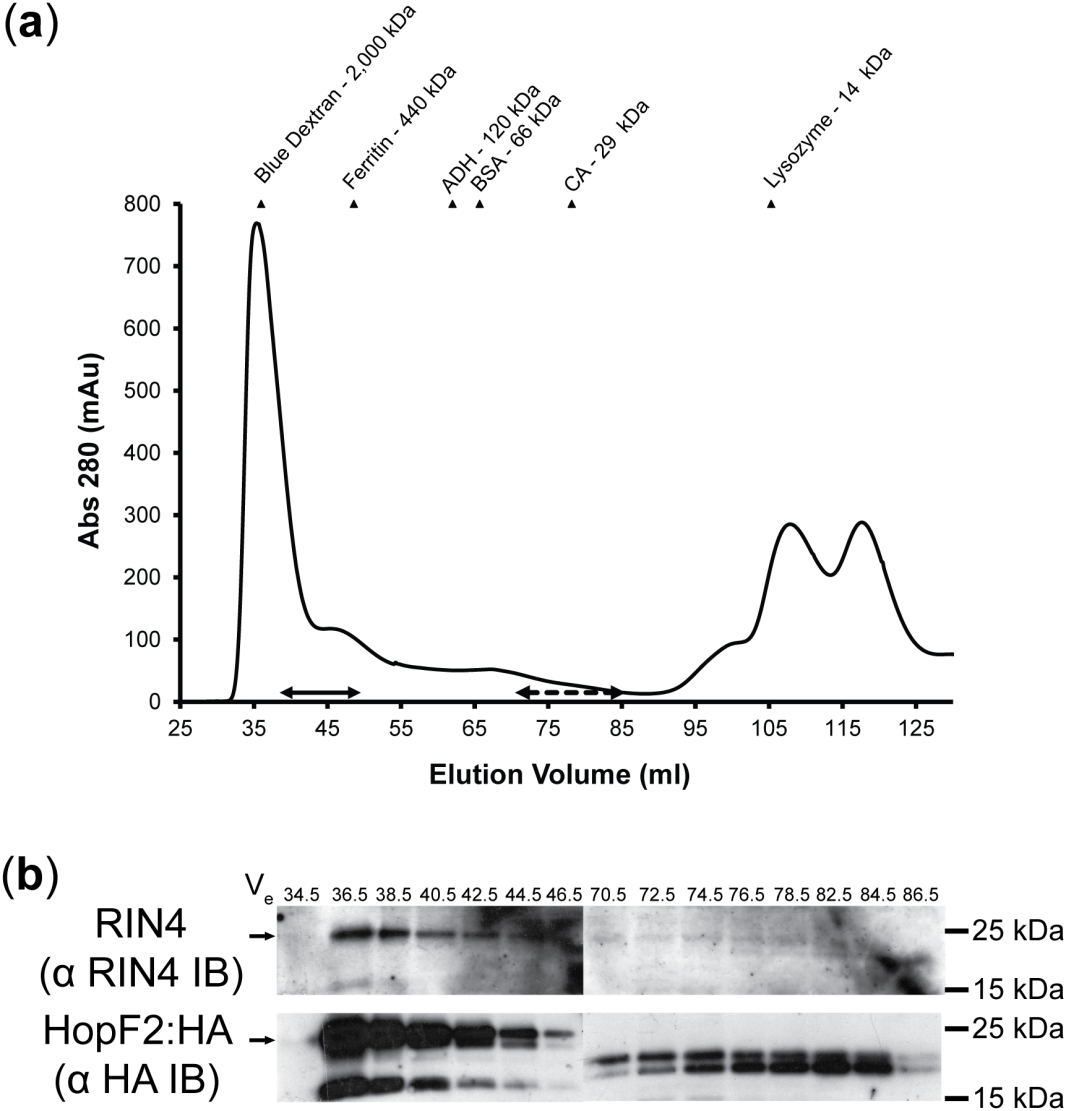
Identification of HopF2/RIN4 complexes by gel filtration chromatography and immunoblotting. Clarified extracts from HopF2:HA overexpressing plants were subjected to gel filtration chromatography on a Sephacryl S-300 HR 16/60 column. Every second fraction from the void volume was resolved by SDS-PAGE and immunoblotted with anti RIN4 immunosera and anti-HA IgG. Bands were visualized with HRP-conjugated secondary anti-bodies and Amersham ECL Advance detection kit. (a) Elution profile of dexamethasone treated Arabidopsis HopF2:HA clarified extract fractionated by gel filtration chromatography. Solid Arrow indicates elution of HA and RIN4 immunoreactive bands. Dashed arrow indicates elution of HA immunoreactive bands alone. Elution volumes of six molecular weight standards are shown as triangles. (b) Immunoblots (IB) showing co-elution of RIN4 and HA immunoreactive bands at high molecular weight. Results are representative of 3 independent replicates. Arrows indicate expected band size for RIN4 (25 kDa) and HopF2 (25 kDa), respectively.

To determine if HopF2 targets high molecular weight (Mr) complexes, protein extracts from Arabidopsis carrying dexamethasone (DEX) inducible HopF2:HA transgene were resolved by gel filtration chromatography using fast protein liquid chromatography (FPLC, Fig. 1a). Fractions were collected from the void volume (V_o_; 36 ml) and every second fraction was analyzed by immunoblot (IB; Fig. 1B) for the presence of HopF2:HA and RIN4, a known interactor of HopF2 [16]. Two peaks containing HopF2:HA were identified with one peak eluting at high molecular weight (Mr; Elution volume (Ve) 36–46 ml, solid arrow Fig. 1) and one eluting around the Mr of monomeric HopF2:HA (Ve 70.5–82.5, dashed arrow Fig. 1). This implies that two pools of HopF2:::HA are present – one associating with high Mr plant protein complexes and one as monomeric HopF2, making gel filtration a viable strategy for enriching HopF2/target protein complexes from HopF2 unbound to targets. Two HopF2 bands were detected in high Mr fractions; one corresponding to the predicted size of the full length protein (∼25 kDa) and one lower Mr product (∼15 kDa). Interestingly, HopF2 in the lower Mr fractions migrated as a doublet between 15 and 25 kDa suggesting that this form of HopF2 is distinct from those found in the high Mr fractions. RIN4 was found to co-elute with HopF2:HA in high Mr fractions, implying RIN4 exists as a member of a high Mr complex. The elution profile of RIN4 was independent of HopF2:HA expression since RIN4 elutes at the column V_0_ in both untreated and DEX treated HopF2:HA transgenic *Arabidopsis* (S1 Figure).

**Figure 2:**
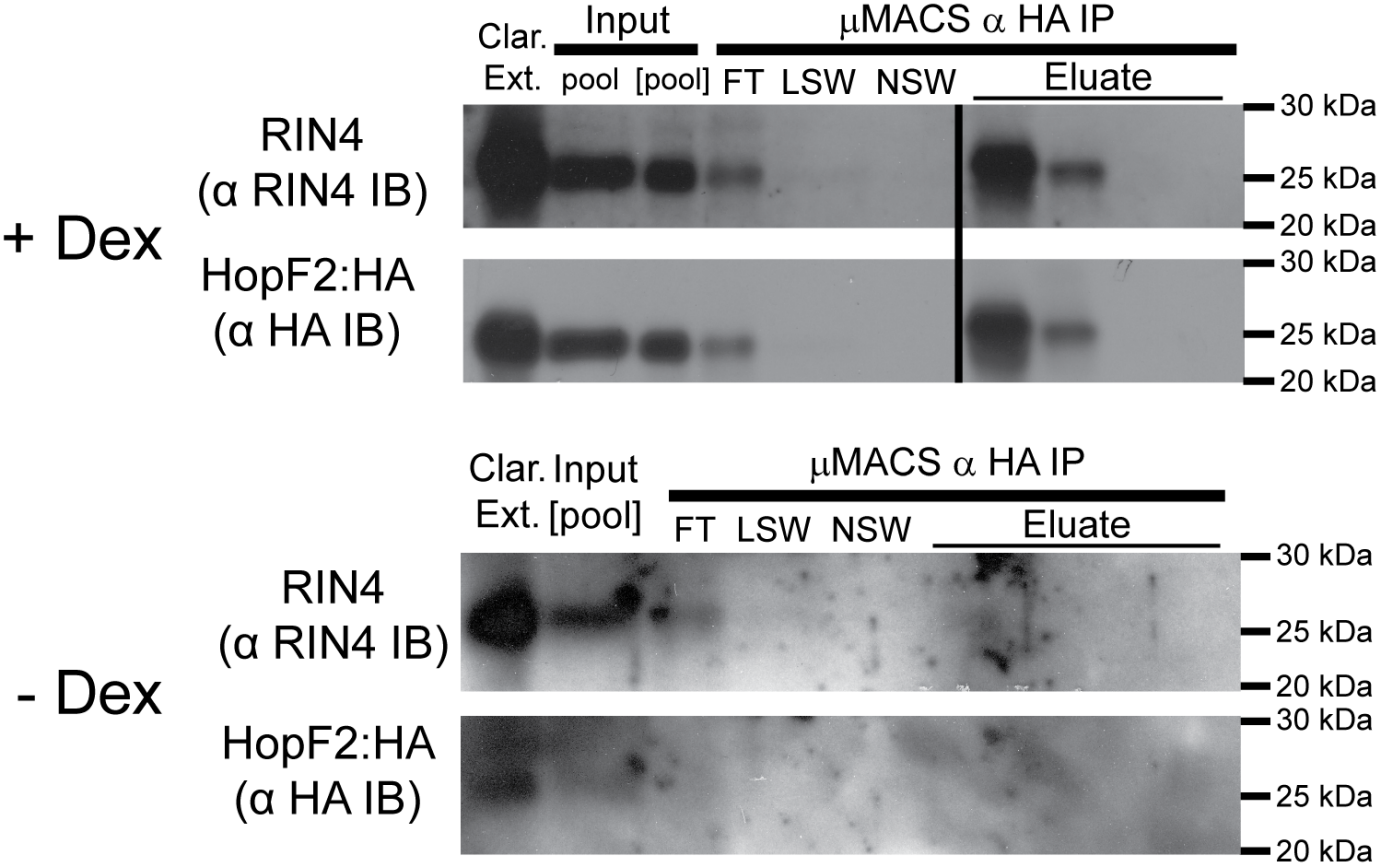
HopF2 and RIN4 co-purifty during immunoaffinity purification from high-molecular weight FPLC fractions. High Mr fractions containing HA immunoreactive (V_e_ 36–46 ml) bands were pooled (input, pool) and concentrated for one hour in a 10,000 Da Mr cutoff concentrator (input, [pool]). Concentrated pooled fractions were incubated with anti-HA magnetic resin (µMACS), immobilized, flow through (FT) collected and resin washed with low salt (LSW), no salt (NSW) buffer then eluted over four fractions with 0.1 M NH_4_OH. Samples were resolved by SDS-PAGE and immunoblotted (IB) with α (RIN4) immunosera and α (HA) IgG. All bands are from the same blot exposure. Blots were cropped to remove molecular weight standards between lanes containing NSW and eluate fractions. Blots of purifications from -DEX tissue were overexposed relative to blots from +DEX purifications. Results are representative of 3 independent replicates.

Next we tested whether HopF2:HA and RIN4 interact in high Mr fractions. We performed immunoaffinity purification of HopF2:HA from concentrated pooled high Mr FPLC fractions ([pool]; Ve 36–46) using anti-(HA) IgG coupled to paramagnetic beads (µMACS α HA IP, Fig. 2). For both RIN4 and HopF2, immunoreactive bands co-migrating with bands from the µMACS α HA IP input ([pool], Fig. 2) were selectively depleted from flow through fraction (FT, Fig. 2) and strongly enriched in fractions eluted from µMACS α HA IP (Eluate, Fig. 2). Washing with low salt (LSW) or no salt (NSW) failed to elute either protein, suggesting a bona fide protein-protein interaction. In contrast, µMACS α HA IP performed on high Mr FPLC fractions from uninduced (–DEX) *Arabidopsis* showed no HopF2:HA reactive bands in input, washes or eluate and no RIN4 immunoreactive bands in µMACS α HA IP eluates despite overexposure of blots (Fig. 2, lower panel). Therefore, isolation of RIN4 was dependent on the presence HopF2:HA, suggesting that HopF2 and RIN4 interact in high Mr complexes isolated from Arabidopsis.

Next we sought to determine what other proteins are present in the high Mr HopF2/RIN4 complex using proteomic methods. Eluates from µMACS α HA IP were analyzed by liquid chromatography tandem mass spectrometry (LC-MS/MS) to determine the identities of proteins co-purified with HopF2:HA. Protein ID by LC-MS/MS of samples eluted with NH_4_OH was confounded by spectra dominated by non-specific contaminants, such as rubisco and light harvesting complex proteins. Therefore, to improve the purity of µMACS α HA IP for protein identification by LC-MS/MS elutions were attempted with HA peptide in phosphate buffered saline (PBS) [36], [37]. Elutions performed with HA peptide in PBS (PBS) produced eluates enriched for HopF2:HA and amenable to proteomic identification (S2 Figure). Three µMACS α HA IP experiments from HopF2:HA induced (+ DEX) and three experiments from uninduced (–DEX) Arabidopsis were analyzed by LC-MS/MS. Proteins identities were assigned using SeQuest to search the UniProt Arabidopsis FASTA database (S1 Table). Proteins were considered as HopF2 complex members if they were identified with at least two unique peptides (p<0.05) in every + DEX HopF2 experiment and zero protein identities in any – DEX experiments (Table 1).

**Table 1:** Proteins of the HopF2 complex identified by mass spectrometry.

AGI	Description	Sample 1^a^	Sample 2^a^	Sample 3^a^
At4g30190	**AHA2 - P-type ATPase** * †	9	4	2
At1g59870	AtPDR8/PEN3 - ABC transporter*	8	9	7
At1g30360	**ERD4** †	7	4	4
At3g53420	PIP2A – Aquaporin**	5	4	3
At3g11130	Clathrin heavy chain	5	3	3
At5G62670	AHA11 - P-type ATPase	3	3	2
At3g08580	ADP/ATP Carrier Protein	2	2	2
At2g45820	**AtREM1.3 Remorin Family Protein** †	2	3	2
At3g01290	**AtHIR2– Hypersensitive Induced Reaction Protein** †	2	4	3
At4g35100	PIP3– Aquaporin**	3	2	2

a Counts are the number of unique peptides identified in each + DEX HopF2 experiment. Protein ID required P<0.01 (Peptide Prophet), minimum two unique peptides in each experiment and zero peptides in any negative control. None of these proteins were identified in any – DEX experiment. Bold text indicates proteins associated with RIN4 or RPS2 protein complexes [29], [39].

*Proteins identified as differentially phosphorylated in response to flg22 [42].

**Proteins identified as differentially phosphorylated in response to ABA [43].

† Proteins enriched in plasma membrane subdomains after flg22 treatment [41].

Our proteomic analysis identified ten proteins as members of a high Mr complex co-purified with HopF2:HA. All are membrane associated proteins, which is consistent with the predicted myristoylation of HopF2 and its membrane localization *in planta* [16], [38]. Although RIN4 was not identified by our mass spectrometry analysis, AHA2 and ERD4 are known members of a RIN4 complex that mediates the closing of stomata in response to *P. syringae* [29]. AtHIR2 is an RPS2 interacting protein identified through proteomic methods [39], [40]. AtHIR2 and AtREM1.3 are thought to play a role in the formation of plasma membrane subdomains in response to pathogen perception [40], [41] while AtPDR8 and AHA2 become phosphorylated in flg22-treated Arabidopsis cell culture [42]. Both PIP2a and PIP3 show increased phosphorylated after ABA treatment [43]. It would therefore appear that HopF2 interacts with a membrane localized complex that is involved in the response to pathogens. In particular, the presence of AHA2 in the HopF2 complex led us to hypothesize that HopF2 can manipulate stomatal immunity.

### HopF2 inhibits stomatal immunity

**Figure 3:**
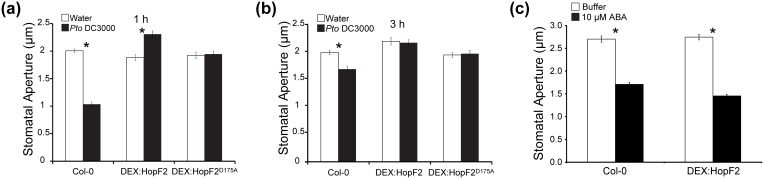
Transgenic expression of HopF2 results in altered stomatal immunity. Four week old Col-0 or transgenic Arabdiopsis expressing HopF2 or HopF2^D175A^ under the control of a dexamethasone inducible promoter were sprayed with 30 µM dexamethasone. Leaf discs were prepared 24 hours after dexamethasone treatment and incubated in water or 1**×**10^8^ CFU suspensions of wild type *Pto* DC3000. Epidermal peels were performed after one (a) or three (b) hours incubation and stomatal aperture was measured. Values are means ± S.E.M of n = 60 stomata from 3**–**5 leaves. Results are representative of 3 independent replicates. Asterisks denote significant differences between means (t-test, p<0.05). (c) ABA treatment induces stomatal closure in transgenic Arabidopsis expressing HopF2. Two week old Col-0 or transgenic Arabdiopsis expressing HopF2 or HopF2^D175A^ under the control of a dexamethasone inducible promoter were sprayed with 30****µM dexamethasone. First and second true leaves were detached one hour after dexamethasone treatment and incubated in buffer overnight then buffer or 10****µM ABA for two hours. Epidermal peels were performed and stomatal aperture was measured. Values are means ± S.E.M of n = 60 stomata from 3**–**5 leaves.

To investigate whether HopF2 can alter stomatal immunity we determined if HopF2 expression can alter the stomatal response to pathogens in Arabidopsis. Leaf discs prepared from Col-0 and transgenic Arabidopsis expressing HopF2:HA were floated in water or bacterial suspensions of *Pto* DC3000. Epidermal peels were produced and used to determine stomatal aperture after 1 and 3 hours (Fig. 3). While treatment with *Pto* DC3000 caused a significant two-fold reduction in stomatal aperture after one hour relative to the water treated control, Arabidopsis expressing HopF2:HA treated with *Pto* DC3000 did not show a reduction in stomatal aperture and instead had significantly wider stomatal apertures than the water control (Fig. 3a). Next, we monitored the stomatal response of transgenic Arabidopsis expressing mutant HopF2^D175A^:HA, which lacks residues required for ADP-ribosyl transferase (ADP-RT) activity [15]. Interestingly, HopF2^ D175A^:HA expressing plants showed no difference between *Pto* DC3000-treated and water-treated controls (Fig. 3b). After three hours, stomatal aperture of *Pto* DC3000 treated plants increased 1.6 fold while plants expressing HopF2:HA and HopF2^ D175A^:HA continued to show no difference to water controls (Fig. 3). Therefore expression of HopF2 or HopF2^D175A^ results in stomata insensitive to PAMP induced stomatal closure, indicative of disabled stomatal immunity. This loss of stomatal immunity was not due to disruption of stomatal function since HopF2-expressing plants still closed stomata in response to ABA treatment (Fig. 3c).

### HopF2 promotes the growth of coronatine-deficient *P. syringae* independently of D175 and RIN4

**Figure 4:**
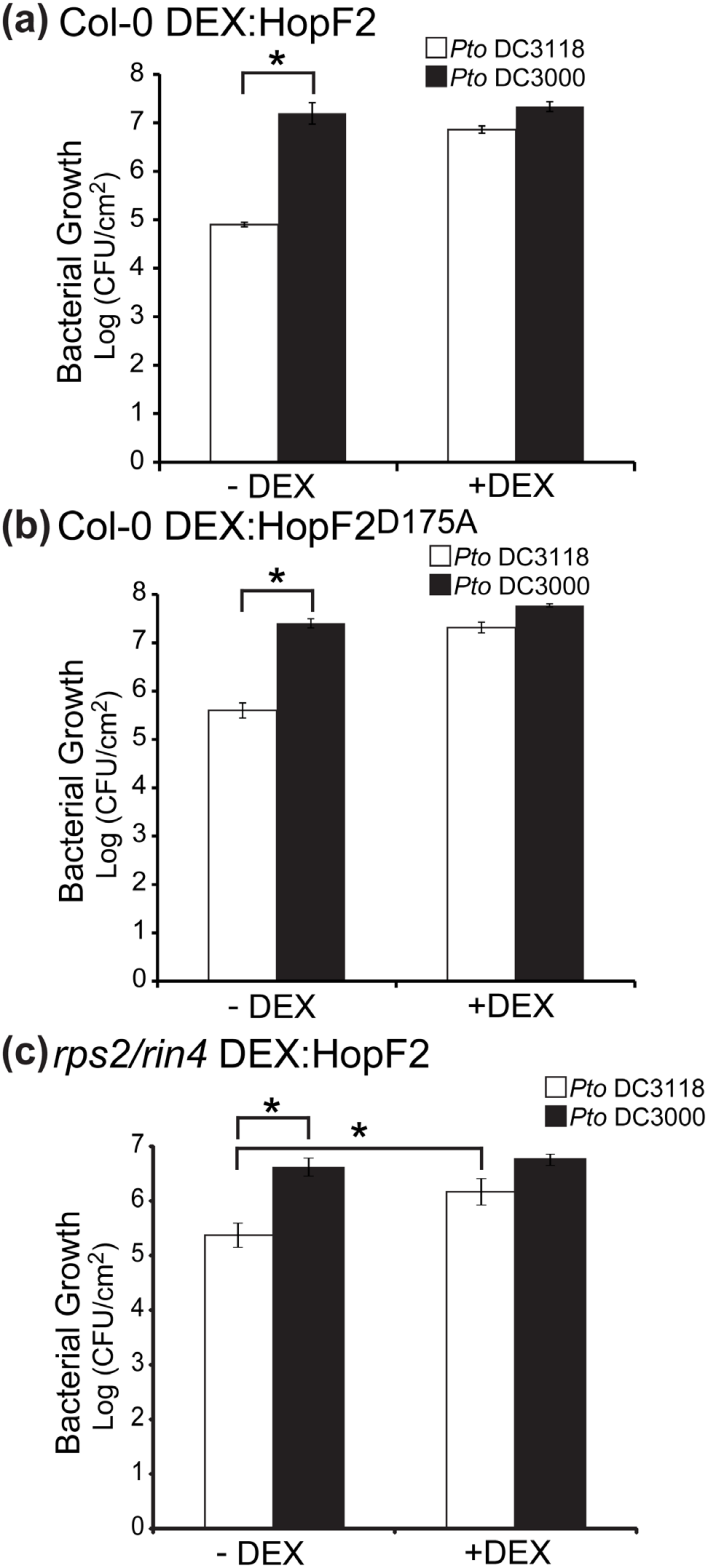
Transgenic expression of HopF2 increases virulence of surface inoculated, coronatine deficient *Pto* DC3118. Four week old transgenic Arabidopsis expressing HopF2:HA under the control of a dexamethasone inducible promoter were sprayed with dexamethasone (+DEX) or water (–DEX). Plants were dipped into 1**×**10^8^ CFU suspensions of wild type *Pto* DC3000 or coronatine deficient *Pto* DC3118. (a) Wild-type HopF2:HA in Col-0 plants. (b) HopF2^D175A^::HA in Col-0 plants. (c) Wild-type HopF2:HA in *rin4/rps2* plants. Bacterial growth curve analyses were performed 3 days post inoculation. Values are means ± S.E.M from n = 12 plants. Asterisks denote significant differences between means (t-test, p<0.05). Results are representative of 3 independent replicates.

To further investigate whether HopF2 can compromise stomatal immunity we investigated whether transgenic expression of HopF2 in Arabidopsis can alter the growth of surface inoculated *P. syringae* (Fig. 4). The phytotoxin coronatine produced by *Pto* DC3000 disables stomatal immunity, preventing stomatal closure in response to bacterial perception [44]. Consequently, *Pto* DC3000 grows well when surface inoculated on Arabidopsis while the coronatine deficient *P. syringae pv.* tomato D3118 (*Pto* DC3118) grows poorly by comparison [2]. If HopF2 can manipulate stomatal immunity, transgenic expression of HopF2 should restore virulence of surface inoculated *Pto* DC3118. Therefore we carried out bacterial *in planta* growth assays by dip inoculation with *Pto* DC3000 and *Pto* DC3118 on +/− DEX HopF2:HA-expressing Arabidopsis (Fig. 4a). After three days of growth in – DEX Arabidopsis, *Pto* DC3000 grew to significantly higher levels than *Pto* DC3118 (Fig. 4a). However, there was no significant difference in growth between *Pto* DC3000 and *Pto* DC3118 in + DEX Arabidopsis expressing HopF2:HA (Fig. 4a). Bacterial growth was also assayed two days after inoculation with identical trends, implying that differences in bacterial growth are not due to apoplastic proliferation late in the time course (S3 Figure). Interestingly, transgenic expression of HopF2^ D175A^:HA also increases the virulence of surface inoculated *Pto* DC3118 (Fig. 4b). Therefore, HopF2 can promote the virulence of coronatine-deficient *P. syringae* independently of the D175 catalytic residue.

To investigate if RIN4 is required for the ability of HopF2 to promote *Pto* DC3118 growth, DEX-inducible HopF2 transgenic plants were generated by crossing the DEX inducible transgene into *rin4/rps2* mutant Arabidopsis plants [24]. Plants were surface inoculated with *Pto* DC3000 or *Pto* DC3118 and growth was measured +/− HopF2 expression. In the absence of HopF2 expression *Pto* DC3000 grew 1.22 logs more than *Pto* DC3118 over three days. Whereas HopF2:HA expression promoted the growth of *Pto* DC3118 and *Pto* DC300 to similar levels. Expression of HopF2:HA also significantly increases the growth of *Pto* DC3118 in *rin4/rps2* plants (white bars, Fig. 4c, t-test, p<0.05). Together, this demonstrates that HopF2 can promote the growth of the coronatine deficient strain *Pto* DC3118 independently of RIN4 (Fig. 4c).

### The HopF2 catalytic residue D175 is differentially required to block PTI responses

**Figure 5:**
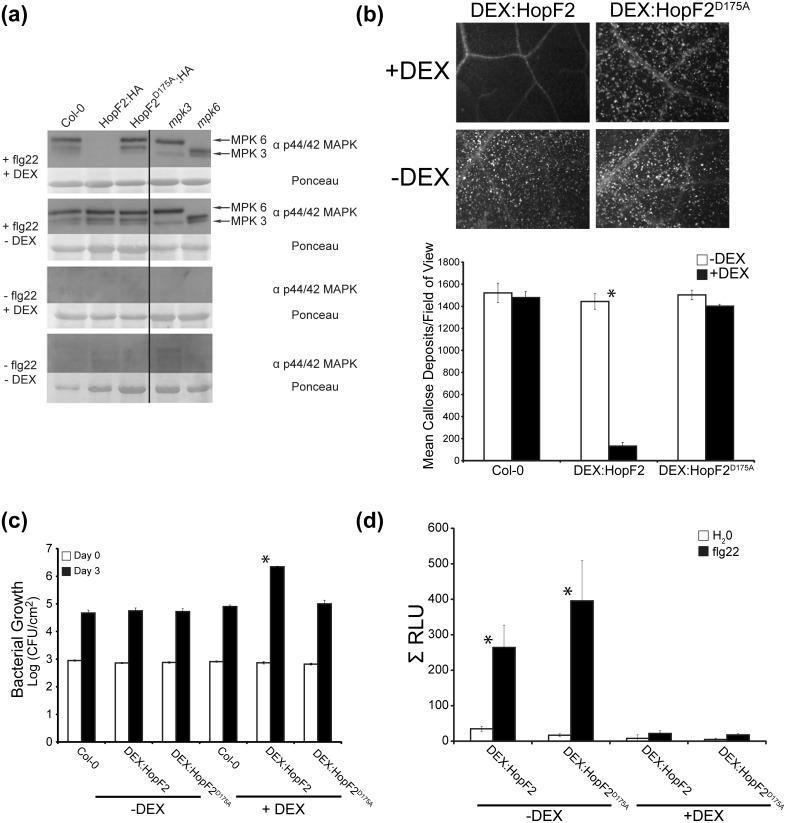
HopF2 inhibits PTI through distinct molecular mechanisms. (a) Transgenic expression of HopF2 subverts MAP kinase activation in Arabidopsis. Eleven-day-old seedlings of Col-0, transgenic Arabidopsis expressing HopF2 and mutant Arabidopsis lacking *mpk3* or *mpk6* were incubated with 3 µM DEX (+DEX) or water (–DEX) 24 h prior to treatment with 1 µM flg22 (+flg22) or water. Samples were collected 20 min after treatment with flg22 (see Methods). Activated MAPKs were detected by immunoblotting using anti-p44/42 MAPK antibody. Mutant Arabidopsis lacking *mpk3* or *mpk6* represent negative controls for the respective MAPK bands. Ponceau staining is presented as a loading control. Results are representative of 5 independent replicates. (b) Transgenic expression of HopF2 inhibits callose deposition triggered by infiltration of *Pto* DC3000Δ*hrcC*. Four week old Col-0 or transgenic Arabidopsis expressing HopF2 were sprayed with 30 µM DEX (+DEX) or water (–DEX) 24 h prior to inoculation. Leaves were stained with aniline blue and visualized 24 h after inoculation of 1×10^8^ cfu/mL of *Pto* DC3000Δ*hrcC*. Callose deposition was quantified using Image J by counting number of papillae in 12 field of views per treatment. Values are means ± SEM from n = 12 leaves. Asterisk denotes statistically significant differences between DEX treatment samples as determined by a pairwise Student’s t-test (P<0.05). Results are representative of 3 independent replicates. (c) Transgenic expression of HopF2 increases growth of syringe inoculated *Pto* DC3000Δ*hrcC.* Four week old Col-0 or transgenic Arabidopsis expressing HopF2 were treated with 30 µM DEX (+DEX) or water (–DEX) immediately after bacterial inoculation (1×10^5^ cfu/ml). Bacterial counts were performed 1 h after inoculation (day 0; filled bars) or 3 days postinoculation (day 3; open bars). Values are means ± SEM from n = 12 leaves. Asterisk denotes statistically significant differences between day 3 samples as determined by a pairwise Student’s t-test (p<0.05). Results are representative of 3 independent replicates. (d) Transgenic expression of HopF2 inhibits flg22 triggered ROS production. Four week old transgenic Arabidopsis expressing HopF2 were sprayed with 30 µM DEX (+DEX) or water (–DEX) 24 h prior to inoculation and leaf discs floated in 2 µM flg22. ROS production was quantified using luminol and horseradish peroxidase with luminescence recorded every two minutes for 60 min. Values are mean sums of total luminescence ± SEM from from n = 6 wells. Asterisk denotes statistically significant differences between DEX treatment samples as determined by a pairwise Student’s t-test (P<0.05).

The ADP-ribosyltransferase activity of HopF2 mediates its virulence function in disabling apoplastic immunity [15], [16]. Further, HopF2 is known to ADP-ribosylate and disable MAP kinases downstream of flg22 perception, as well as RIN4 [15]. Consistent with this we were able to demonstrate that transgenic expression of HopF2:HA prevents phosphorylation of MPK3/6 after flg22 treatment in a catalytic residue-dependent manner since HopF2^ D175A^:HA did not prevent MPK3/6 phosphorylation (Fig. 5a). In addition, D175 was required for the ability of HopF2 to block flg22-induced callose deposition and promote the growth of the *Pto* DC3000Δ*hrcC* mutant which lacks a functional T3SS and is unable to suppress PTI (Fig. 5b, c). Perception of flg22 is also known to trigger an increase in the production of oxidative intermediates that help signal stomatal closure [10]. We therefore sought to determine if expression of HopF2 can alter the flg22 induced oxidative burst using a luminol-dependent chemiluminescence assay. Transgenic HopF2:HA and HopF2^ D175A^:HA plants were induced with dexamethasone 24 hours before conducting the ROS assay. Application of flg22 strongly induced ROS production in Col-0 and transgenic plants without DEX treatment, whereas expression of either HopF2:HA or HopF2^ D175A^:HA blocked ROS production. Therefore, HopF2 can block the PTI-associated ROS burst independently of the catalytic residue D175 (Fig. 5d).

## Discussion

In this study we demonstrated that HopF2 can block stomatal immunity independently of its ADP-ribosyltransferase activity [15]. This activity was independent of RIN4 and was not due to a general disruption of stomatal function since HopF2 did not block ABA-induced stomatal closure (Fig. 3c).

We used a proteomic approach to identify a HopF2 associated protein complex in Arabidopsis. This complex contained two RIN4-associated proteins (AHA2 and ERD4), and one RPS2-associated protein (AtHIR2), supporting our previous report that HopF2 targets RIN4 [16], [29], [39]. Our method failed to identify RIN4, RPM1 or RPS2 directly as members of this complex, although RIN4 could be detected by immunoblotting (Fig. 1). Although RPS2 was previously detected in purified RIN4 complexes, RPM1 was not [29]. Proteomic investigation of RPS2 interacting proteins also failed to reliably identify RIN4, despite being readily detectable by immunoblot [39]. The small size and high basic amino acid content of RIN4 may make it difficult to detect by standard proteomic methods. A failure to identify the RIN4 interacting NLR-proteins RPM1 or RPS2 might also be due to low NLR-protein expression levels relative to RIN4 or ATPase proteins and/or the presence of RIN4 complexes not associated with these NLR-proteins [29]. Our approach may also decrease the possibility of discovering NLR-proteins since our bait (HopF2) does not directly interact with these proteins, and requires RIN4 to “bridge” interactions with NLR-proteins in a tertiary complex. Nevertheless, our proteomic approach supports that HopF2 forms part of a high Mr RIN4-containing complex *in planta*.

Two populations of HopF2 were identified from our size exclusion chromatography analysis. One population elutes in high Mr fractions and contained RIN4, whereas another population elutes in low Mr fractions corresponding to the size of monomeric HopF2 (Fig. 1). The high Mr HopF2 population mirrored the distribution of RIN4 and spanned molecular weights of 300 kDa to approximately 1000 kDa. This population may represent one complex or multiple large complexes. Since these complexes are likely to membrane associated, their size may reflect complexes held together by proteins and/or lipids that are maintained during the extraction procedure [41]. Directed binary interaction maps will help resolve which proteins from these complexes can directly interact versus those that may co-localize to the same membrane microdomains.

It is interesting to note that the HopF2 eluting in lower Mr fractions following size exclusion chromatography was smaller than HopF2 from high Mr fractions according to their SDS-PAGE migration rates (Fig. 1b). High Mr fractions contained HopF2 that migrated at the expected 25 kDa with a lower band at about 15 kDa, whereas HopF2 from low Mr fractions migrated as a doublet between 15 and 25 kDa, with no band corresponding to predicted full length HopF2. Since the HA tag used for immunoblotting is translationally fused to the C-terminus, the lower molecular weight HopF2 likely represents an N-terminal truncation. An N-terminal truncation of HopF2 would remove the myristoylation sequence which is required for its membrane localization [38]. As such, this may represent a population of HopF2 that is not membrane associated *in planta* thereby expanding its range of targets to include soluble proteins.

Our proteomics approach led us to hypothesize and demonstrate that HopF2 can block stomatal immunity. Transgenic expression of HopF2 can inhibit stomatal closing triggered by *P. syringae* (Fig. 3) and improves the virulence of surface inoculated coronatine deficient *P. syringae* strain *Pto* DC3118 (Fig. 4). It is possible that some virulence promotion of surface inoculated *Pto* DC3118 is mediated by HopF2’s ability to inhibit apoplastic immunity (Fig. 5a–c) as opposed to increased stomatal invasion. However, HopF2’s ability to promote the virulence of syringe inoculated *P. syringae* depends on the catalytic residue D175, as well as the host protein RIN4 [16]. Consistent with this, transgenic expression of HopF2^D175A^ does not increase the growth of syringe inoculated mutant *P. syringae* lacking a functional T3SS (Fig. 5c). In contrast, transgenic expression of HopF2^D175A^ or HopF2 in *rin4/rps2* Arabidopsis promotes the virulence of surface inoculated *Pto* DC3118 (Fig. 4b, c). Therefore, HopF2 can inhibit stomatal immunity independent of the molecular mechanisms required for its inhibition of apoplastic immunity.

Two early PTI signalling events involved in stomatal immunity, MAP kinase activation and ROS production, were differentially inhibited by transgenic expression of HopF2 (Fig. 5). Inhibition of MAP kinase activation induced by the flagellar PAMP flg22 was dependent on the catalytic D175 residue, whereas inhibition of ROS production was not. MAPK activation and ROS production represent independent, early PTI signalling events induced by flg22 perception [45]. The ADP-RT dependent inhibition of MAP kinase signalling by HopF2 likely involves its direct targeting of MAPKKs or the PRR co-receptor BAK1 [15], [17].

A salient question that remains is what protein is targeted by HopF2 to suppress stomatal immunity? It is unlikely to be RIN4 since HopF2 can promote *Pto* DC3118 growth in *rps2/rin4* plants (Fig. 4c). It is also unlikely to be the H+-ATPases AHA1/2 since their disruption would likely alter ABA-responsiveness [46]. The ability of HopF2 to block ROS production independently of its ADP-RT activity would indicate that it targets a signalling component that influences ROS production but not MAPK activation in order to block stomatal immunity. Potential targets include the NADPH oxidase RBOHD, the RLCKs BSK1 and PBL1, and/or the aspartate oxidase FIN4 [47]–[52].

If HopF2 does target a protein involved in the early bifurcation of PRR-signaling in order to suppress stomatal immunity, what is its mode of action? The crystal structure of HopF1 revealed that it adopts a mushroom-like structure with a head subdomain that possess the ADP-RT catalytic residues and a “stalk” subdomain with no obvious structural similarity to known enzymes. Both subdomains contained surface-exposed residues that were important for both virulence and avirulence activities in bean [14]. HopF2 is predicted to adopt a similar structure to HopF1_Pph1449B_ (Phyre2, identity = 54%, confidence = 100.0) [53]. Since the suppression of stomatal immunity by the HopF2 is independent of the ADP-RT catalytic activity of the “head” domain, it is possible that residues of the “stalk” are required for this function, in which case HopF2 would represent a bifunctional effector similar to the *Pseudomonas aeruginosa* T3SE ExoT. ExoT has an N-terminal GTPase-activating protein (GAP) domain and a C-terminal ADP-RT domain [54], [55]. Although the predicted head and stalk subdomains of HopF2 would not be separable as C- and N-terminal fragments, it is possible that HopF2 has evolved a mushroom-like structure to accommodate two independent functions. An important next step to characterizing the ADP-RT-independent function will involve the identification of HopF2 residues required for its ability to block stomatal immunity. In addition, it remains to be determined whether HopF2 carries out this second function directly in guard cells or influences stomatal immunity from non-stomatal cells. There is evidence that the T3SS functions during epiphytic growth [56]. However, it remains to be determined whether T3SEs are injected into guard cells and whether they can function in guard cells, for example, when expressed from guard cell specific promoters.

Overall, our results support the hypothesis that HopF2 is a bifunctional T3SE. One function involves its ADP-RT activity that is required for its ability to block PTI-activated MAP kinase signaling and to promote post-invasive apoplastic growth in a RIN4-dependent manner [15], [16]. A second function is ADP-RT independent and involves the suppression of PRR-triggered stomatal-immunity in a RIN4-independent manner. Therefore HopF2 has separable PTI suppressing functions and represents an appealing probe to study signal branching following PRR activation.

## Supporting Information

S1 Figure **RIN4 exists in a high Mr complex independent of HopF2 expression.** Clarified extracts from induced (+DEX) and induced (–DEX) plants expressing HopF2:HA under the control of a dexamethasone inducible promoter were subjected to gel filtration chromatography on a Sephacryl S-300 HR 16/60 column. Every second fraction from the void volume was resolved by SDS-PAGE and immunoblotted with anti RIN4 immunosera and anti HA IgG.
(TIF)

S2 Figure **HopF2 is successfully eluted with HA peptide in PBS.** High molecular weight FPLC fractions containing HA immunoreactive (V_e_ = 36–46 ml) bands were pooled and concentrated for one hour in a 10,000 Mr cutoff concentrator. Concentrate was incubated with anti HA magnetic resin. HA resin was immobilized and complexes were eluted over three bed volumes of 250 µg/ml HA peptide in PBS. Samples were resolved by SDS-PAGE and immunoblotted with anti RIN4 immunosera and anti HA IgG.
(TIF)

S3 Figure **Transgenic expression of HopF2 increases virulence of surface inoculated, coronatine deficient** ***Pto*** **DC3118 two days after dip inoculation.** Transgenic Arabidopsis expressing HopF2:HA under the control of a dexamethasone inducible promoter were dipped into 1×10^8^ CFU suspensions of wild type *Pto* DC3000 or coronatine deficient *Pto* DC3118. (a) Wild-type HopF2:HA in Col-0 plants. (b) HopF2^D175A^::HA in Col-0 plants. (c) Wild-type HopF2:HA in *rin4/rps2* plants. Bacterial growth curve analyses were performed 2 days post inoculation. Values are means ± S.E.M from n = 12 plants. Asterisks denote significant differences between means (t-test, p<0.05). Results are representative of 3 independent replicates.
(TIF)

S1 Table **All proteins identified in HopF2 µMACS α HA IP experiments.** All protein identified by LC-MS/MS analysis of µMACS HA IP eluate from 3 dexamethasone treated (+DEX) and 3 untreated (CTL) samples of transgenic Arabidopsis expressing HopF2 from a dexamethasone inducible promoter.
(XLSX)

## References

[1] Morris CE, Monteil CL, Berge O (2013) The life history of Pseudomonas syringae: linking agriculture to earth system processes. Annu Rev Phytopathol 51:85–104 doi:10.1146/annurev-phyto-082712-102402. 23663005

[2] Melotto M, Underwood W, Koczan J, Nomura K, He SY (2006) Plant stomata function in innate immunity against bacterial invasion. Cell 126:969–980 doi:10.1016/j.cell.2006.06.054. 16959575

[3] Nicaise V, Roux M, Zipfel C (2009) Recent advances in PAMP-triggered immunity against bacteria: pattern recognition receptors watch over and raise the alarm. Plant Physiol 150:1638–1647 doi:10.1104/pp.109.139709. 19561123 PMC2719144

[4] Sawinski K, Mersmann S, Robatzek S, Böhmer M (2013) Guarding the green: pathways to stomatal immunity. Mol Plant Microbe Interact 26:626–632 doi:10.1094/MPMI-12-12-0288-CR. 23441577

[5] Zheng X, Spivey N, Zeng W, Liu P, Zheng Q, et al (2012) Coronatine Promotes Pseudomonas syringae Virulence in Plants by Activating a Signaling Cascade that Inhibits Salicylic Acid Accumulation. Cell host … 11:587–596 doi:10.1016/j.chom.2012.04.014.Coronatine. PMC3404825 22704619

[6] Geng X, Jin L, Shimada M, Kim MG, Mackey D (2014) The phytotoxin coronatine is a multifunctional component of the virulence armament of Pseudomonas syringae. Planta. doi:10.1007/s00425-014-2151-x. PMC4228168 25156488

[7] Deslandes L, Rivas S (2012) Catch me if you can: bacterial effectors and plant targets. Trends Plant Sci 17:644–655 doi:10.1016/j.tplants.2012.06.011. 22796464

[8] Lewis JD, Guttman DS, Desveaux D (2009) The targeting of plant cellular systems by injected type III effector proteins. Semin Cell Dev Biol 20:1055–1063 doi:10.1016/j.semcdb.2009.06.003. 19540926

[9] Freeman BC, Beattie GA (2009) Bacterial growth restriction during host resistance to Pseudomonas syringae is associated with leaf water loss and localized cessation of vascular activity in Arabidopsis thaliana. Mol Plant Microbe Interact 22:857–867 doi:10.1094/MPMI-22-7-0857. 19522568

[10] Lozano-Durán R, Bourdais G, He SY, Robatzek S (2014) The bacterial effector HopM1 suppresses PAMP-triggered oxidative burst and stomatal immunity. New Phytol 202:259–269 doi:10.1111/nph.12651. 24372399

[11] Gimenez-Ibanez S, Boter M, Fernández-Barbero G, Chini A, Rathjen JP, et al (2014) The bacterial effector HopX1 targets JAZ transcriptional repressors to activate jasmonate signaling and promote infection in Arabidopsis. PLoS Biol 12:e1001792 doi:10.1371/journal.pbio.1001792. 24558350 PMC3928049

[12] Tsiamis G, Mans JW, Hockenhull R, Jackson RW, Sesma A, et al (2000) Cultivar-specifc avirulence and virulence functions assigned to avrPphF in Pseudomonas syringae pv. phaseolicola, the cause of bean halo-blight disease. EMBO J 19:3204–3214. 10880434 10.1093/emboj/19.13.3204 PMC313945

[13] Hou S, Mu R, Ma G, Xu X, Zhang C, et al (2011) Pseudomonas syringae pv. phaseolicola effector HopF1 inhibits pathogen-associated molecular pattern-triggered immunity in a RIN4-independent manner in common bean (Phaseolus vulgaris). FEMS Microbiol Lett 323:35–43 doi:10.1111/j.1574-6968.2011.02356.x. 22092678

[14] Singer AU, Desveaux D, Betts L, Chang JH, Nimchuk Z, et al (2004) Crystal structures of the type III effector protein AvrPphF and its chaperone reveal residues required for plant pathogenesis. Structure 12:1669–1681 doi:10.1016/j.str.2004.06.023. 15341731

[15] Wang Y, Li J, Hou S, Wang X, Li Y, et al (2010) A Pseudomonas syringae ADP-ribosyltransferase inhibits Arabidopsis mitogen-activated protein kinase kinases. Plant Cell 22:2033–2044 doi:10.1105/tpc.110.075697. 20571112 PMC2910962

[16] Wilton M, Subramaniam R, Elmore J, Felsensteiner C, Coaker G, et al (2010) The type III effector HopF2Pto targets Arabidopsis RIN4 protein to promote Pseudomonas syringae virulence. Proc Natl Acad Sci U S A 107:2349–2354 doi:10.1073/pnas.0904739107. 20133879 PMC2836640

[17] Zhou J, Wu S, Chen X, Liu C, Sheen J, et al (2014) The Pseudomonas syringae effector HopF2 suppresses Arabidopsis immunity by targeting BAK1. Plant J 77:235–245 doi:10.1111/tpj.12381. 24237140 PMC4224013

[18] Aoyama T, Chua NH (1997) A glucocorticoid-mediated transcriptional induction system in transgenic plants. Plant J 11:605–612. 9107046 10.1046/j.1365-313x.1997.11030605.x

[19] Mindrinos M, Katagiri F, Yu G, Ausubel F (1994) The A. thaliana disease resistance gene RPS2 encodes a protein containing a nucleotide-binding site and leucine-rich repeats. Cell 78:1089–1099. 7923358 10.1016/0092-8674(94)90282-8

[20] Mackey D, Holt BF, Wiig A, Dangl JL (2002) RIN4 interacts with Pseudomonas syringae type III effector molecules and is required for RPM1-mediated resistance in Arabidopsis. Cell 108:743–754. 11955429 10.1016/s0092-8674(02)00661-x

[21] Liu Y, Zhang S (2004) Phosphorylation of 1-aminocyclopropane-1-carboxylic acid synthase by MPK6, a stress-responsive mitogen-activated protein kinase, induces ethylene biosynthesis in. Plant Cell 16:3386–3399 doi:10.1105/tpc.104.026609.1. 15539472 PMC535880

[22] Tsuda K, Mine A, Bethke G, Igarashi D, Botanga CJ, et al (2013) Dual regulation of gene expression mediated by extended MAPK activation and salicylic acid contributes to robust innate immunity in Arabidopsis thaliana. PLoS Genet 9:e1004015 doi:10.1371/journal.pgen.1004015. 24348271 PMC3861249

[23] Wang H, Ngwenyama N, Liu Y, Walker JC, Zhang S (2007) Stomatal development and patterning are regulated by environmentally responsive mitogen-activated protein kinases in Arabidopsis. Plant Cell 19:63–73 doi:10.1105/tpc.106.048298. 17259259 PMC1820971

[24] Mackey D, Belkhadir Y, Alonso JM, Ecker JR, Dangl JL (2003) Arabidopsis RIN4 is a target of the type III virulence effector AvrRpt2 and modulates RPS2-mediated resistance. Cell 112:379–389. 12581527 10.1016/s0092-8674(03)00040-0

[25] Lewis JD, Abada W, Ma W, Guttman DS, Desveaux D (2008) The HopZ family of Pseudomonas syringae type III effectors require myristoylation for virulence and avirulence functions in Arabidopsis thaliana. J Bacteriol 190:2880–2891 doi:10.1128/JB.01702-07. 18263728 PMC2293245

[26] Moore RA, Starratt AN, Ma S, Cuppels DA, Al MET (1989) Identification of a chromosomal region required for biosynthesis of the phytotoxin coronatine by Pseudomonas syringae pv. tomato. Can J Microbiol 35:910–917.

[27] Ma SW, Morris VL, Cuppels DA (1991) Characterization of a DNA region required for the production of the phytotoxin coronatine by Pseudomo nas syringae pv. tomato. Mol Plant Microbe Interact 4:69–74.

[28] Deng W, Preston G, Collmer A, Chang C (1998) Characterization of the hrpC and hrpRS Operons of Pseudomonas syringae Pathovars Syringae, Tomato, and Glycinea and Analysis of the Ability of hrpF, hrpG, hrcC, hrpT, and hrpV Mutants To Elicit the Hypersensitive Response and Disease in Plants. J Bacteriol 180:1523–4531. 10.1128/jb.180.17.4523-4531.1998 PMC107463 9721291

[29] Liu J, Elmore JM, Fuglsang AT, Palmgren MG, Staskawicz BJ, et al (2009) RIN4 functions with plasma membrane H+-ATPases to regulate stomatal apertures during pathogen attack. PLoS Biol 7:e1000139 doi:10.1371/journal.pbio.1000139. 19564897 PMC2694982

[30] Angers S, Thorpe CJ, Biechele TL, Goldenberg SJ, Zheng N, et al (2006) The KLHL12-Cullin-3 ubiquitin ligase negatively regulates the Wnt-beta-catenin pathway by targeting Dishevelled for degradation. Nat Cell Biol 8:348–357 doi:10.1038/ncb1381. 16547521

[31] Nesvizhskii AI, Keller A, Kolker E, Aebersold R (2003) A Statistical Model for Identifying Proteins by Tandem Mass Spectrometry abilities that proteins are present in a sample on the basis. Anal Chem 75:4646–4658. 14632076 10.1021/ac0341261

[32] Adam L, Somerville SC (1996) Adam and Somerville - Genetic characterization of five powdery mildew disease resistance loci in Arabidopsis thalian.pdf. Plant J 9:341–356. 8919911 10.1046/j.1365-313x.1996.09030341.x

[33] Hauck P, Thilmony R, He SY (2003) A Pseudomonas syringae type III effector suppresses cell wall-based extracellular defense in susceptible Arabidopsis plants. Proc Natl Acad Sci U S A 100:8577–8582 doi:10.1073/pnas.1431173100. 12817082 PMC166271

[34] Abràmoff M, Magalhaes P, Ram S. (2004) Image processing with ImageJ. Biophotonics Int 11:36–42.

[35] Felix G, Duran JD, Volko S, Boller T (1999) Plants have a sensitive perception system for the most conserved domain of bacterial flagellin. Plant J 18:265–276. 10377992 10.1046/j.1365-313x.1999.00265.x

[36] Sowa ME, Bennett EJ, Gygi SP, Harper JW (2009) Defining the human deubiquitinating enzyme interaction landscape. Cell 138:389–403 doi:10.1016/j.cell.2009.04.042. 19615732 PMC2716422

[37] Behrends C, Sowa ME, Gygi SP, Harper JW (2010) Network organization of the human autophagy system. Nature 466:68–76 doi:10.1038/nature09204. 20562859 PMC2901998

[38] Robert-Seilaniantz A, Shan L, Zhou J-M, Tang X (2006) The Pseudomonas syringae pv. tomato DC3000 type III effector HopF2 has a putative myristoylation site required for its avirulence and virulence functions. Mol Plant Microbe Interact 19:130–138 doi:10.1094/MPMI-19-0130. 16529375

[39] Qi Y, Katagiri F (2009) Purification of low-abundance Arabidopsis plasma-membrane protein complexes and identification of candidate components. Plant J 57:932–944 doi:10.1111/j.1365-313X.2008.03736.x. 19000159

[40] Qi Y, Tsuda K, Nguyen L V, Wang X, Lin J, et al (2011) Physical association of Arabidopsis hypersensitive induced reaction proteins (HIRs) with the immune receptor RPS2. J Biol Chem 286:31297–31307 doi:10.1074/jbc.M110.211615. 21757708 PMC3173095

[41] Keinath NF, Kierszniowska S, Lorek J, Bourdais G, Kessler S a, et al (2010) PAMP (pathogen-associated molecular pattern)-induced changes in plasma membrane compartmentalization reveal novel components of plant immunity. J Biol Chem 285:39140–39149 doi:10.1074/jbc.M110.160531. 20843791 PMC2998143

[42] Benschop JJ, Mohammed S, O’Flaherty M, Heck AJR, Slijper M, et al (2007) Quantitative phosphoproteomics of early elicitor signaling in Arabidopsis. Mol Cell Proteomics 6:1198–1214 doi:10.1074/mcp.M600429-MCP200. 17317660

[43] Kline KG, Barrett-Wilt G a, Sussman MR (2010) In planta changes in protein phosphorylation induced by the plant hormone abscisic acid. Proc Natl Acad Sci U S A 107:15986–15991 doi:10.1073/pnas.1007879107. 20733066 PMC2936636

[44] Zeng W, He SY (2010) A prominent role of the flagellin receptor FLAGELLIN-SENSING2 in mediating stomatal response to Pseudomonas syringae pv tomato DC3000 in Arabidopsis. Plant Physiol 153:1188–1198 doi:10.1104/pp.110.157016. 20457804 PMC2899927

[45] Macho AP, Zipfel C (2014) Plant PRRs and the Activation of Innate Immune Signaling. Mol Cell 54:263–272 doi:10.1016/j.molcel.2014.03.028. 24766890

[46] Merlot S, Leonhardt N, Fenzi F, Valon C, Costa M, et al (2007) Constitutive activation of a plasma membrane H(+)-ATPase prevents abscisic acid-mediated stomatal closure. EMBO J 26:3216–3226 doi:10.1038/sj.emboj.7601750. 17557075 PMC1914098

[47] Nühse TS, Bottrill AR, Jones AME, Peck SC (2007) Quantitative phosphoproteomic analysis of plasma membrane proteins reveals regulatory mechanisms of plant innate immune responses. Plant J 51:931–940 doi:10.1111/j.1365-313X.2007.03192.x. 17651370 PMC2156193

[48] Zhang J, Shao F, Li Y, Cui H, Chen L, et al (2007) A Pseudomonas syringae effector inactivates MAPKs to suppress PAMP-induced immunity in plants. Cell Host Microbe 1:175–185 doi:10.1016/j.chom.2007.03.006. 18005697

[49] Ranf S, Eschen-Lippold L, Pecher P, Lee J, Scheel D (2011) Interplay between calcium signalling and early signalling elements during defence responses to microbe- or damage-associated molecular patterns. Plant J 68:100–113 doi:10.1111/j.1365-313X.2011.04671.x. 21668535

[50] Segonzac C, Feike D, Gimenez-Ibanez S, Hann DR, Zipfel C, et al (2011) Hierarchy and roles of pathogen-associated molecular pattern-induced responses in Nicotiana benthamiana. Plant Physiol 156:687–699 doi:10.1104/pp.110.171249. 21478366 PMC3177268

[51] Feng F, Yang F, Rong W, Wu X, Zhang J, et al (2012) A Xanthomonas uridine 5′-monophosphate transferase inhibits plant immune kinases. Nature 485:114–118 doi:10.1038/nature10962. 22504181

[52] Macho AP, Boutrot F, Rathjen JP, Zipfel C (2012) Aspartate oxidase plays an important role in Arabidopsis stomatal immunity. Plant Physiol 159:1845–1856 doi:10.1104/pp.112.199810. 22730426 PMC3425217

[53] Kelley LA, Sternberg MJE (2009) Protein structure prediction on the Web: a case study using the Phyre server. Nat Protoc 4:363–371 doi:10.1038/nprot.2009.2. 19247286

[54] Krall R, Schmidt G, Aktories K, Barbieri T (2000) Pseudomonas aeruginosa ExoT Is a Rho GTPase-Activating Protein. Infect Immun 68. doi:10.1128/IAI.68.10.6066-6068.2000.Updated. PMC101576 10992524

[55] Sun J, Barbieri JT (2003) Pseudomonas aeruginosa ExoT ADP-ribosylates CT10 regulator of kinase (Crk) proteins. J Biol Chem 278:32794–32800 doi:10.1074/jbc.M304290200. 12807879

[56] Lee J, Teitzel GM, Munkvold K, del Pozo O, Martin GB, et al (2012) Type III secretion and effectors shape the survival and growth pattern of Pseudomonas syringae on leaf surfaces. Plant Physiol 158:1803–1818 doi:10.1104/pp.111.190686. 22319072 PMC3320187

