# Supplementary figures and images for: The Pseudomonas syringae Type III Effector HopF2 Suppresses Arabidopsis Stomatal Immunity
Source: PLoS One. 2014 Dec 11;9(12):e114921. doi: 10.1371/journal.pone.0114921 (PMC4263708; doi:10.1371/journal.pone.0114921)

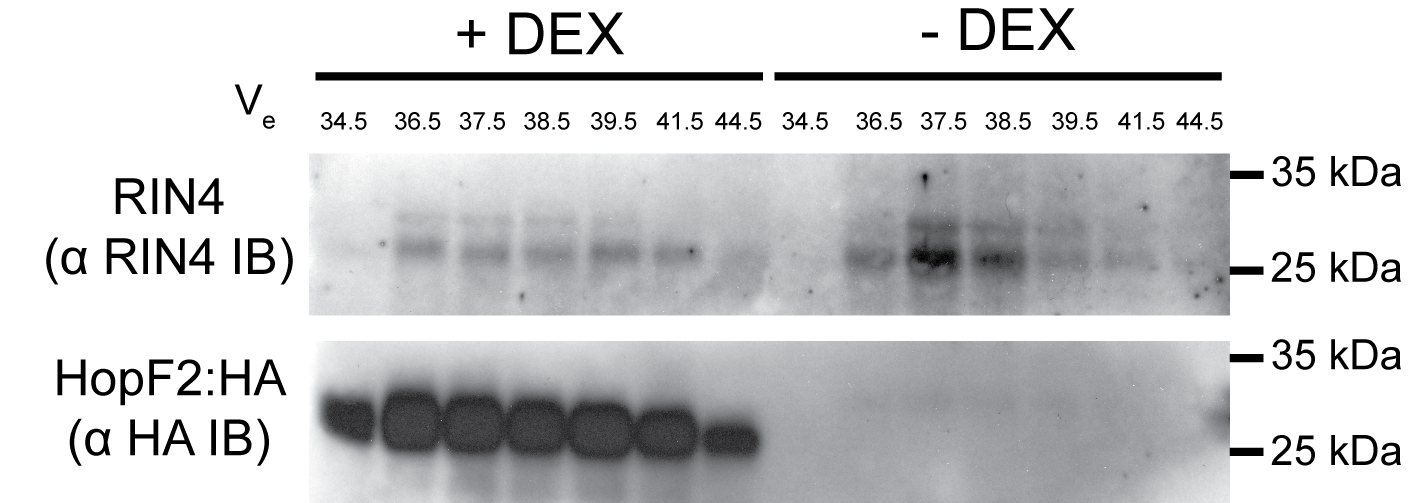

Supplement: S1 Figure — RIN4 exists in a high Mr complex independent of HopF2 expression. Clarified extracts from induced (+DEX) and induced (–DEX) plants expressing HopF2:HA under the control of a dexamethasone inducible promoter were subjected to gel filtration chromatography on a Sephacryl S-300 HR 16/60 column. Every second fraction from the void volume was resolved by SDS-PAGE and immunoblotted with anti RIN4 immunosera and anti HA IgG. (TIF) [file pone.0114921.s001.tif]

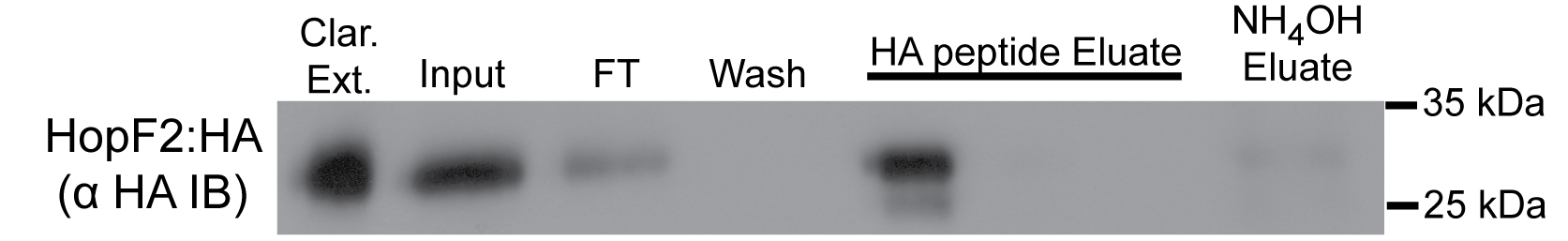

Supplement: S2 Figure — HopF2 is successfully eluted with HA peptide in PBS. High molecular weight FPLC fractions containing HA immunoreactive (Ve = 36–46 ml) bands were pooled and concentrated for one hour in a 10,000 Mr cutoff concentrator. Concentrate was incubated with anti HA magnetic resin. HA resin was immobilized and complexes were eluted over three bed volumes of 250 µg/ml HA peptide in PBS. Samples were resolved by SDS-PAGE and immunoblotted with anti RIN4 immunosera and anti HA IgG. (TIF) [file pone.0114921.s002.tif]

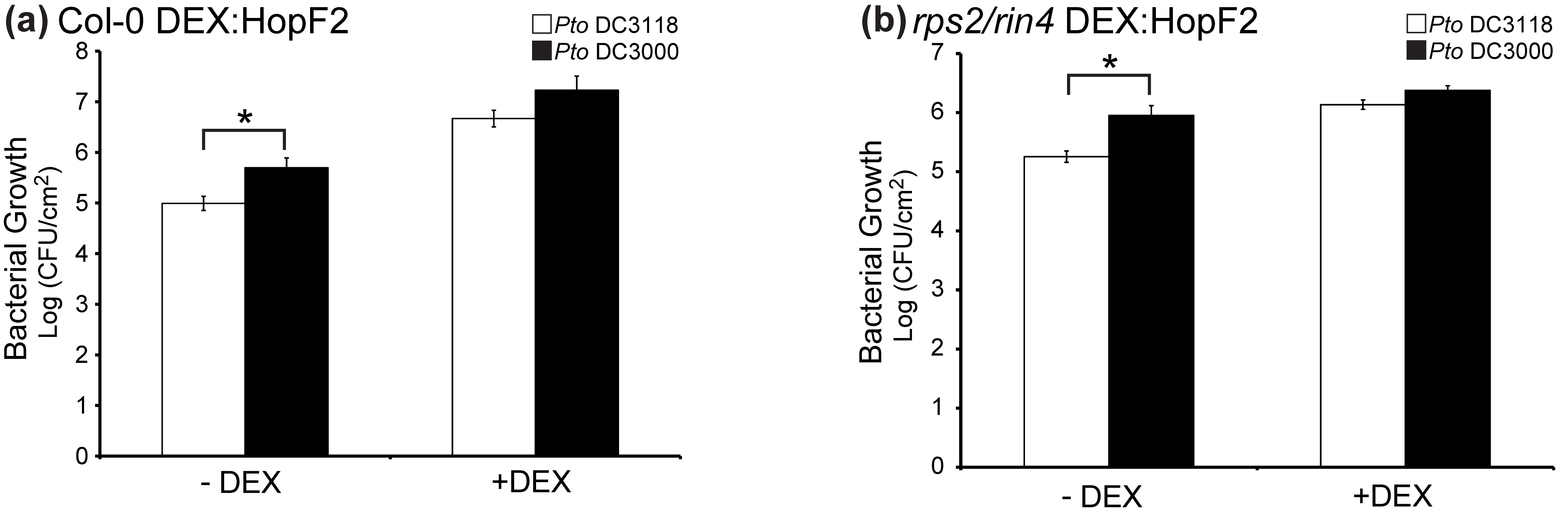

Supplement: S3 Figure — Transgenic expression of HopF2 increases virulence of surface inoculated, coronatine deficient Pto DC3118 two days after dip inoculation. Transgenic Arabidopsis expressing HopF2:HA under the control of a dexamethasone inducible promoter were dipped into 1×10^8^ CFU suspensions of wild type Pto DC3000 or coronatine deficient Pto DC3118. (a) Wild-type HopF2:HA in Col-0 plants. (b) HopF2D175A::HA in Col-0 plants. (c) Wild-type HopF2:HA in rin4/rps2 plants. Bacterial growth curve analyses were performed 2 days post inoculation. Values are means ± S.E.M from n = 12 plants. Asterisks denote significant differences between means (t-test, p<0.05). Results are representative of 3 independent replicates. (TIF) [file pone.0114921.s003.tif]
